# Convulsive Seizure as a Presenting Symptom of Extreme Anaemia: A Case Report

**DOI:** 10.7759/cureus.110234

**Published:** 2026-06-04

**Authors:** See Ming C Wong, Chi Yuen Cheung

**Affiliations:** 1 Medicine, Queen Elizabeth Hospital Hong Kong, Hong Kong, HKG

**Keywords:** acute kidney injury, extreme anaemia, myelodysplastic syndrome, pure red cell aplasia (prca), seizure

## Abstract

Extreme anaemia (haemoglobin <2g/dL) is rarely encountered, and the clinical manifestation of this entity remains unclear. A woman in her 60s who enjoyed good past health presented with a two-week history of generalized malaise and then a sudden onset of convulsive seizure. Blood tests revealed haemoglobin of 1.7g/dL with acute kidney injury and elevated liver enzymes and creatine kinase. She was subsequently diagnosed with myelodysplastic syndrome with erythroid hypoplasia. After receiving a supportive blood transfusion, she had significant improvement in both clinical and biochemical parameters. Our case illustrates that extreme anaemia can present with seizure and multiple organ dysfunction secondary to tissue ischaemia.

## Introduction

In our body, the amount of oxygen supply to an organ is dependent on arterial oxygen saturation, cardiac output, and haemoglobin level. Decreased oxygen delivery due to low haemoglobin level was termed anaemic hypoxia. In addition to acute profound blood loss, other clinical conditions, including infections, cancers, haemopoietic disorders, and malnutrition, can also cause anaemia. Clinical symptoms often depend on the severity of anaemia. Patients commonly develop shortness of breath, tachycardia, dizziness, and generalized malaise [1]. Since extreme anaemia (haemoglobin <2g/dL) is rarely encountered nowadays, the clinical manifestation of this entity remains unclear [2]. Theoretically, extreme anaemia can cause low oxygen delivery to vital organs, leading to a severe mismatch between oxygen supply and demand and subsequently tissue ischaemia and end-organ damage. The most commonly affected organs include the brain, heart, liver, and kidneys, resulting in a variety of clinical conditions, such as neurological deficits, myocardial infarction, acute liver injury, and acute kidney injury (AKI). Here we report a patient who presented with convulsive seizure, liver impairment, and AKI, who was found to have extreme anaemia with hemoglobin 1.7g/dL. She was subsequently diagnosed with myelodysplastic syndrome (MDS) with marked erythroid hypoplasia.

## Case presentation

**Figure 1:**
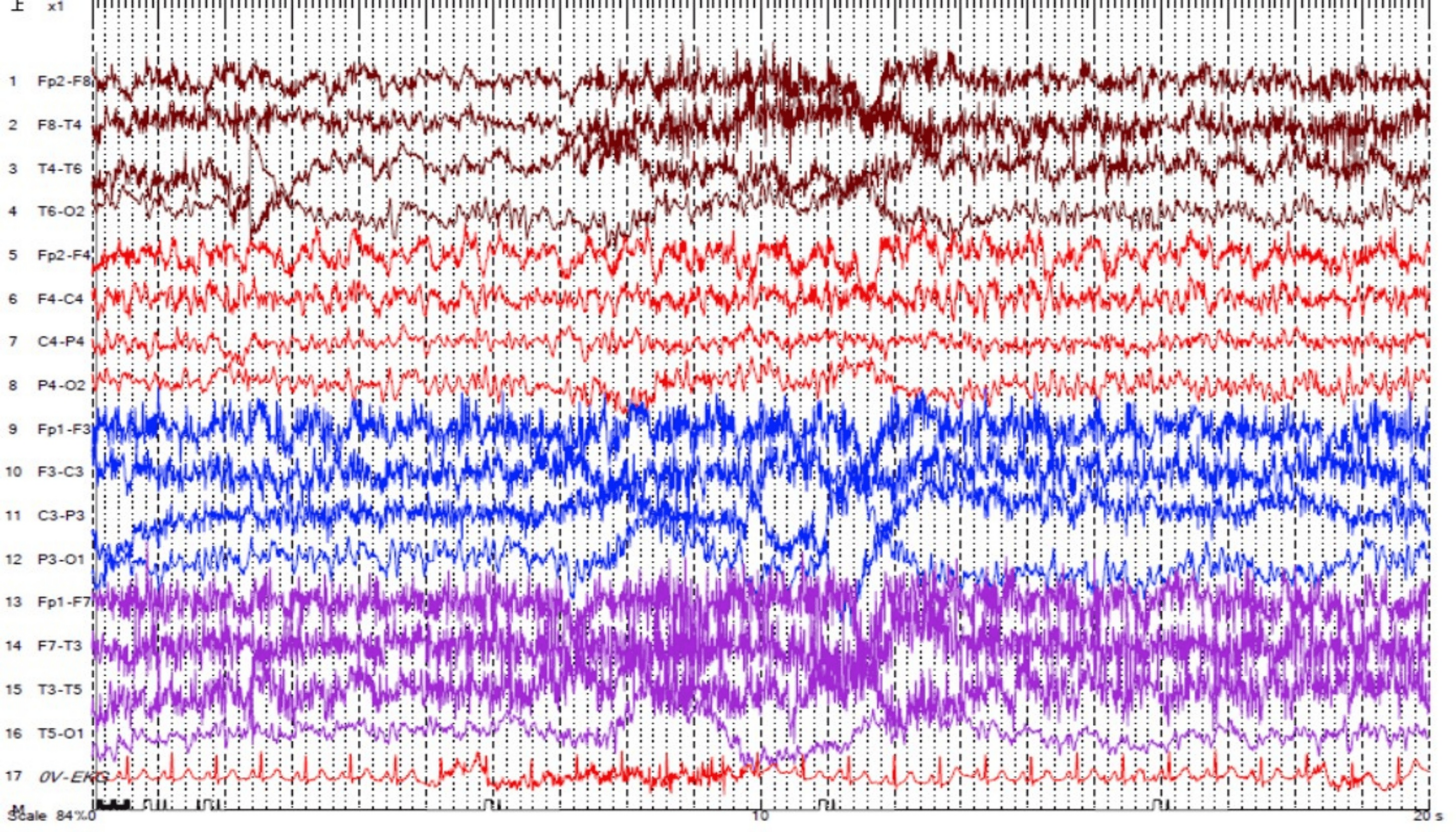
Electroencephalogram did not reveal any significant abnormality

A 60-year-old woman, who enjoyed good past health, complained of malaise for two weeks. She was hospitalized due to the sudden onset of generalized tonic-clonic convulsive seizure for two minutes, followed by postictal drowsiness. There were neither bleeding symptoms nor a substance abuse history. Her blood pressure was 108/56 mmHg with a pulse rate of 100 beats per minute. There was no fever. Physical examination showed marked pallor. There was no lymphadenopathy and hepatosplenomegaly. There was also no focal neurological sign. Blood tests showed severe macrocytic anaemia, with haemoglobin 1.7g/dL, mean cell volume 121.7fL, white blood cell count 19x10^9^/L(neutrophil 11.8x10^9^/L, lymphocyte 5.2x10^9^/L, monocyte 1.8x10^9^/L, eosinophil 0.3x10^9^/L), platelet 605x10^9^/L, and reticulocyte count 1%. The vitamin B12 level, folate level, and iron profile were normal. Haptoglobin level was not low, and the direct Coombs test was negative. Peripheral blood smear did not reveal any blast cells. The liver enzymes were elevated (Table 1). Both hepatitis serology and autoimmune markers were negative. The serum creatinine was 116 µmol/L (estimated glomerular filtration rate 48 ml/min), and creatine kinase (CK) was 1374 IU/L. The lactate level was elevated, being 2.4 mmol/L. Chest X-ray was clear. Computerized tomography (CT), including the brain, thorax, and abdomen, was unremarkable. Lumbar puncture was done, and the cerebrospinal fluid (CSF) was clear. The opening pressure was normal, and CSF workup did not reveal evidence of central nervous system (CNS) infection. The electroencephalogram (EEG) did not show any epileptiform discharge (Figure 1).

**Table 1:** Change of laboratory results before and two weeks after blood transfusion MCV: mean corpuscular volume; ALT: alanine transaminase; AST: aspartate aminotransferase; GGT: gamma-glutamyltransferase; LDH: lactate dehydrogenase; CK: creatinine kinase.

	Before blood transfusion	Two weeks after blood transfusion	Reference interval
Hemoglobin (g/dL)	1.7	9.2	11.7-14.9
White cell count (X 10^9^/L)	19.0	6.7	3.7-9.2
Platelet (X 10^9^/L)	605	354	145-370
MCV (fL)	121.7	89.8	82.0-97.0
Reticulocyte count (%)	1.0	0.9	0.5-2.0
Serum potassium (mmol/l)	3.4	4.1	3.4-5.0
Serum creatinine (umol/L)	116	64	45-84
Serum albumin (g/L)	36	32	33-48
Serum bilirubin (umol/L)	45	9	<27
Serum ALT (IU/L)	875	47	<47
Serum AST (IU/L)	667	25	<35
Serum GGT (IU/L)	103	N.A.	6-42
Serum LDH (IU/L)	1626	232	110-210
Serum CK (IU/L)	1374	70	26-192

**Figure 2:**
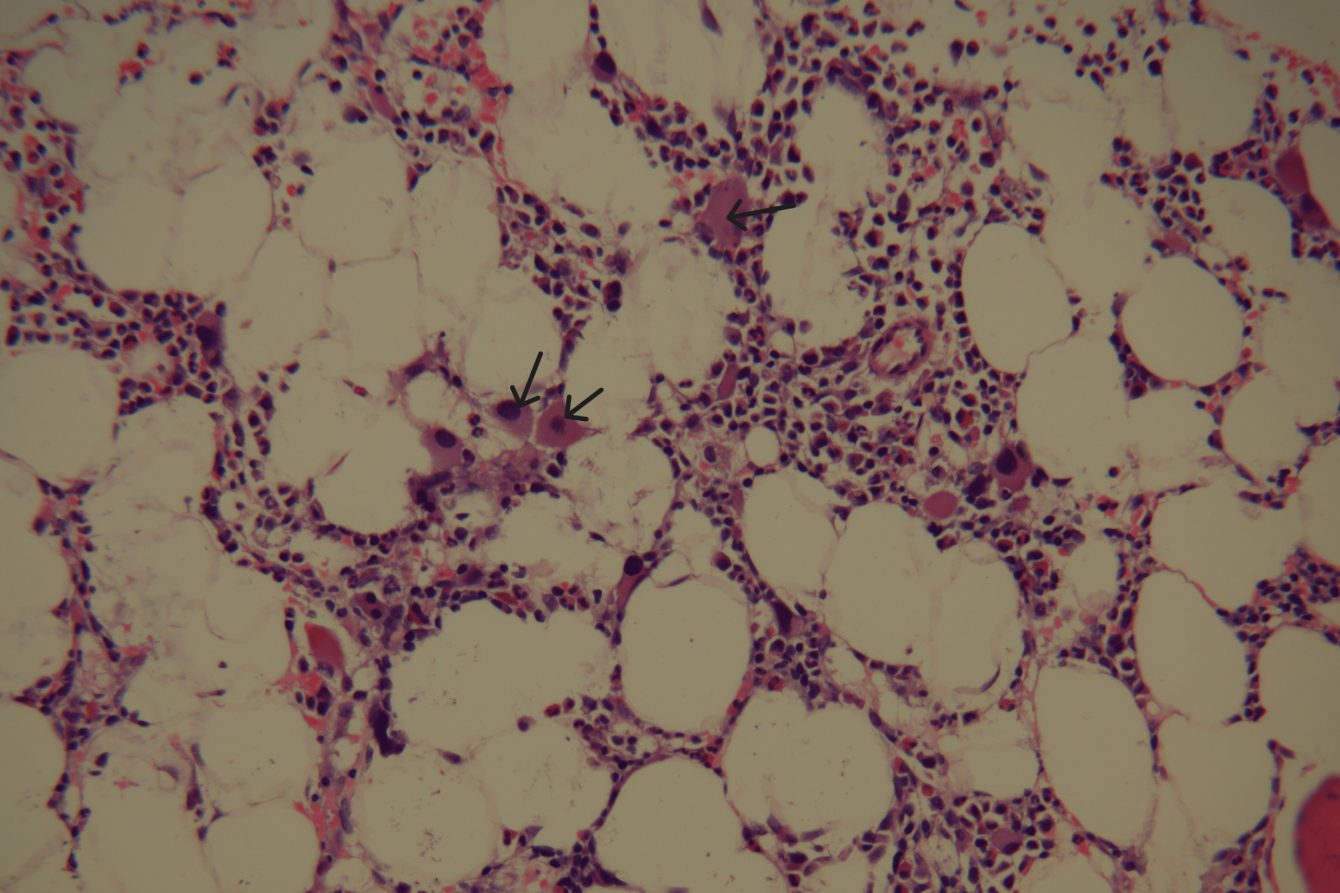
Bone marrow trephine biopsy (H&E stain) showing erythroid hypoplasia and megakaryocytic hyperplasia with dysmegakaryopoiesis (arrows)

Bone marrow examination showed marked erythroid hypoplasia (erythroid precursors 3%), mildly increased granulopoiesis (mild left-shift with full maturation), no excessive blasts (1%), and megakaryocytic hyperplasia with dysmegakaryopoiesis (Figure 2). 

**Figure 3:**
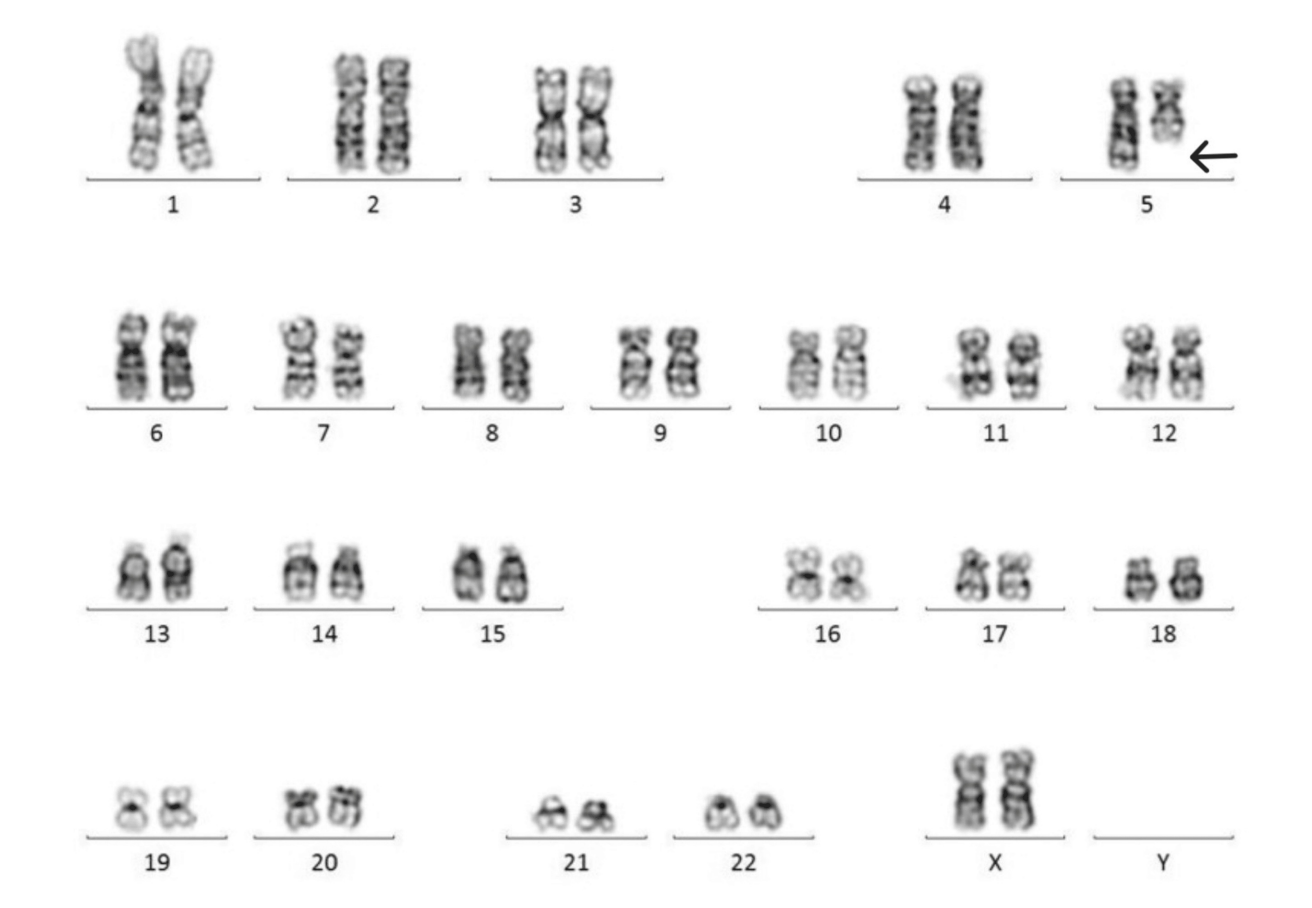
Cytogenetics confirmed heterozygous deletion of 5q (arrow)

Cytogenetics and fluorescence in situ hybridization analysis confirmed heterozygous deletion of 5q and translocation involving 7q material without loss of 7q (Figure 3). 

Serology for parvovirus B19 was negative. A one-week course of cefotaxime 1 gram daily was empirically started on admission due to the presence of neutrophilia. Septic workup, including blood and urine culture, came back to be negative. The blood white cell count normalized 4 days after the commencement of antibiotics. Blood transfusion was also given immediately after hospitalization, and the target hemoglobin level was kept above 8g/dL. Her general condition gradually improved. There were no more seizures. Her biochemical profile, including liver enzymes, serum creatinine, lactate, and CK levels, all normalized within two weeks (Table 1). She continued to receive treatment for MDS in the clinic after discharge.

## Discussion

Our patient was found to have extreme macrocytic anaemia, neutrophilia, and thrombocytosis. Based on the bone marrow examination, cytogenetics, and fluorescence in situ hybridization (FISH) analysis, the final diagnosis of our patient was MDS with del (5q) and erythroid hypoplasia [3]. The neutrophilia was probably related to the presence of concurrent infection, as the white blood cell count normalized after the course of antibiotics. Although uncommon, thrombocytosis (>450 × 10^9^/L) was noticed in 20% of patients with MDS with del(5q) [4]. Extreme anaemia has rarely been reported in the literature. The majority of these patients presented with generalized weakness, exhaustion, shortness of breath, and abdominal pain. However, uncommon presentations such as transient neurological deficit have also been found in patients with extreme anaemia [5]. As shown in a retrospective cohort study, anaemia could lead to dysfunction in multiple organs, including the heart, brain, and kidneys [6]. Our patient had a history of malaise before the development of a seizure. She was also found to have AKI, deranged liver function, and elevated CK. For the cause of convulsive seizure, epilepsy, stroke, and CNS infection were excluded by the EEG, cerebral CT, and CSF findings. Moreover, she did not have any toxin exposure. It was hypothesized that her seizure attack could be a result of transient hypoxic injury to brain tissue, and the increase in oxygen consumption during seizure might further aggravate her hypoxic condition. Seizure as a manifestation of acute severe anaemia has been described in a hemodialysis patient with profound blood loss, and anoxic brain injury was shown in neuroimaging of this patient [7]. In addition, the development of AKI, elevated liver enzymes, and CK in our patient could also be explained by ischaemic injury to the tissues in the kidneys, liver, and muscles, respectively. She did not have any clinical features suggestive of prerenal AKI. Since her renal function had already normalized after the correction of anaemia, there was no further workup for her AKI. The lack of hepatotoxic agents, negative hepatitis serology, and autoimmune markers suggested that ischaemia might be the cause of hepatitis in her. In fact, there was a report showing that ischaemic hepatitis can occur in extreme anaemia without significant reduction in cardiac output or severe hypoxaemia [8]. However, the exact mechanism leading to the convulsive seizure and the other clinical features in our patient could not be proven in this case report. Besides supportive blood transfusion, definite treatment of the underlying MDS is necessary in our patient. Lenalidomide, a synthetic immunomodulatory thalidomide derivative, is highly effective in MDS patients with del(5q) [9]. 

## Conclusions

Extreme anaemia may cause ischaemic tissue injury in multiple organs. Apart from common symptoms such as malaise and weakness, we should also be aware of some potential severe manifestations of anaemia, including convulsive seizure, AKI, and liver derangement. Supportive blood transfusion can help to improve the symptoms and biochemical abnormalities before investigating the underlying cause of anaemia. The long-term prognosis, however, depends on the treatment of the underlying disease rather than the degree of the acute anaemia.
